# Simulation-based multi-criteria decision making: an interactive method with a case study on infectious disease epidemics

**DOI:** 10.1007/s10479-021-04321-8

**Published:** 2021-10-12

**Authors:** Fabian Dunke, Stefan Nickel

**Affiliations:** Institute of Operations Research, Discrete Optimization and Logistics, Karlsruhe Institute of Technology, Kaiserstr. 12, 76131 Karlsruhe, Germany

**Keywords:** Multi-criteria decision making, Sensitivity analysis, Simulation-based decision making, Infectious disease epidemic simulation

## Abstract

Whenever a system needs to be operated by a central decision making authority in the presence of two or more conflicting goals, methods from multi-criteria decision making can help to resolve the trade-offs between these goals. In this work, we devise an interactive simulation-based methodology for planning and deciding in complex dynamic systems subject to multiple objectives and parameter uncertainty. The outline intermittently employs simulation models and global sensitivity analysis methods in order to facilitate the acquisition of system-related knowledge throughout the iterations. Moreover, the decision maker participates in the decision making process by interactively adjusting control variables and system parameters according to a guiding analysis question posed for each iteration. As a result, the overall decision making process is backed up by sensitivity analysis results providing increased confidence in terms of reliability of considered decision alternatives. Using the efficiency concept of Pareto optimality and the sensitivity analysis method of Sobol’ sensitivity indices, the methodology is then instantiated in a case study on planning and deciding in an infectious disease epidemic situation similar to the 2020 coronavirus pandemic. Results show that the presented simulation-based methodology is capable of successfully addressing issues such as system dynamics, parameter uncertainty, and multi-criteria decision making. Hence, it represents a viable tool for supporting decision makers in situations characterized by time dynamics, uncertainty, and multiple objectives.

## Introduction

A simulation model is an executable model of a real world system which can be utilized to elicit information about future system behavior (Law and Kelton 2000). Therefore, a crucial function of simulation is to support decision makers with prescriptive guidance concerning future system trajectories. However, the role of simulation as part of model-driven decision support systems is rather perceived as that of a separated assistance system (Power and Sharda 2007). Neither a systematic integration nor a formalized feedback mechanism between these tools for computerized decision making is yet established on a general level, but rather in problem-specific settings (Hopfe 2009; Heilala et al. 2010; Mahdavi et al. 2010; Kadri et al. 2014a; Fanti et al. 2015). Apart from the technological perspective, many real world systems exhibit a number of challenging characteristics: Uncertainty in system parameters, complexity in terms of size and relations between entities, involvement of multiple conflicting objectives, large degree of time dynamic system elements (Anderson 1999; Grösser 2017). As a major advantage, simulation inherently addresses the latter three of these challenges: No global analytical formula is required to describe the overall system behavior; instead, individual specifications of system elements collectively cause a system trajectory to occur (Banks 2010; Cassandras and Lafortune 2010). Concerning the consideration of two or more goals in a setting of multi-criteria decision making, simulation benefits from the possibility to track an arbitrary number of performance measures with virtually no computational cost. Nonetheless, uncertainty handling cannot be tackled by simulation as a standalone tool. Therefore, various frameworks have been proposed in the form of parameter variation, sensitivity analysis, or simulation optimization. According to Dellino and Meloni (2015), these approaches are intrinsically related to each other when simulation is seen as an underlying method to evaluate the quality of specific system parameter and control variable settings.

As an application, consider an infectious disease epidemic comparable to the 2020 coronavirus pandemic. Political decision makers have to weigh health, societal, and economic impacts of their decisions influencing the course of the epidemic, e.g., by shutting down the economy temporarily (Raboisson and Lhermie 2020; Malmir and Zobel 2021; Pamučar et al. 2020). This setting is coined by the need for sophisticated decision making amidst uncertainty, time dynamics, and multiple objectives. Since the population to be considered is large, a system dynamics simulation is adequate to model infections within the population over time. The model can then be deployed in a feedback loop with sensitivity analysis methods to evaluate alternative courses of action. As the output of the entire process, decision makers learn about the sensitivity of system parameters (which cannot be changed suddenly, but maybe present opportunities for changes in the long run) and control variables (which allow for immediately influencing the course of events), guiding them towards an informed overall decision. In this sense, decision makers gain confidence in their decisions and develop a better overall understanding of the system with respect to the different objectives.

**Fig. 1:**
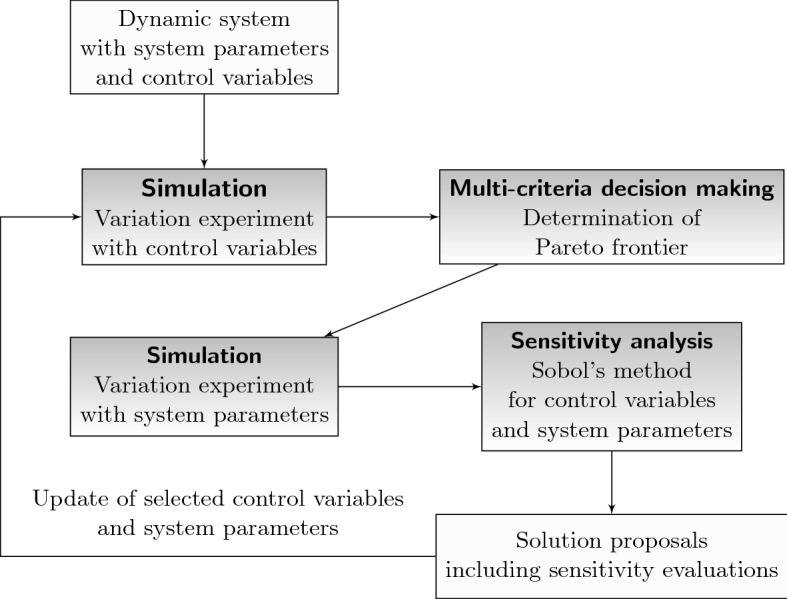
Algorithmic outline for simulation-based multi-criteria decision making

The main contribution of the paper consists of a holistic methodological outline for the analysis of decision making tasks in real-world complex dynamic systems subject to uncertainty, time dynamics, and multiple objectives. As shown in Fig. 1, this is achieved through a combination of simulation models, sensitivity analysis, and multi-criteria decision making addressing the mentioned challenges jointly. Used over several iterations, the updating step leads to increased confidence with respect to the final decision. This is particularly favorable in applications exhibiting uncertainty in critical parameters and strong sensitivity of model outputs to these parameters. As a result, the decision maker is not only in a position to make a well-informed decision once, but also understands the system more deeply acquiring knowledge to be used in similar future situations. Methodologically, this conclusion can be drawn from the detailed sensitivity analysis which is deployed upon system parameters and control variables throughout the overall process. Moreover, the method represents an adjustable approach to sequential decision making under uncertainty as it allows to adaptively warm-start a simulation model with the currently observed system state. In this sense, not only parameter uncertainty is considered, but also decision adaptability. Finally, the case study on infectious disease epidemics provides a template for using the methodology in a concrete practical problem setting. We remark that the goal of the paper is not to provide a perfect simulation model for the 2020 coronavirus outbreak, but to develop a framework supporting decision makers in similar situations amenable to simulation-based decision making.

The remainder of the paper is organized as follows: Sect. 2 discusses research on topics of methodological interest (such as multi-criteria decision making, simulation and optimization, sensitivity analysis) and on topics of practical interest (such as epidemics simulation). The main contribution of the paper is found in Sect. 3: Combining approaches from multi-criteria decision making and sensitivity analysis, we introduce an interactive simulation-based methodology for planning and deciding in complex dynamic systems which are characterized by uncertainty and multiple objectives. The case study in Sect. 4 yields experimental evidence on how the methodology can support decision makers in an infectious disease epidemic. Finally, Sect. 5 presents challenging areas for future research.

## Related work

We discuss existing literature on the different topics combined in this paper. The presentation has a converging character with respect to these topics in order to show how simulation, multi-criteria decision making, sensitivity analysis are interrelated.

### Simulation and optimization

Simulation models reproduce the behavior of a real world system in a computerized model to gain insight into ruling causes and effects relations of the system. Depending on the system, different modeling paradigms with different abstraction levels are used (Siebers et al. 2010; Sumari et al. 2013; Tako and Robinson 2018). Discrete-event models proceed based on a detailed resolution of events. System dynamics are appropriate when large populations are considered in an aggregated perspective. Recently, agent-based models have gained importance. They allow to model individual behavior and interactions as the autonomous driving force of the simulation. This is in contrast to discrete-event models and system dynamics with their bottom-up and top-down perspectives, respectively (Macal 2010). Multi-method simulation mixes these approaches when different types of granularity are required (Brailsford et al. 2010). Frameworks to streamline multi-method model development are introduced by Morgan et al. (2017), Mykoniatis (2015). Applications of simulation modeling relevant to this paper are found for health care including infectious disease treatment in Djanatliev and German (2013), Gunal (2012), Mustafee et al. (2013), Viana et al. (2014).

The most frequent combination of simulation and optimization comprises the optimization of parameters of a simulation model with respect to the model output. System parameters are considered as strategic control variables, and simulation is used as an evaluation function of a parameter setting (Fu 1994). Since not all parameter settings may be evaluated, optimization becomes necessary, ranging from meta-heuristics to simulation metamodeling (Carson and Maria 1997). Reviews focus on different parameter domains (continuous vs. discrete), leading to methods from ranking and selection to stochastic approximation; metaheuristics are explicitly discussed to facilitate global optimization (Amaran et al. 2016; Tekin and Sabuncuoglu 2004). However, these methods consider single objectives. Methods for parameter optimization in simulations with multiple objectives are missing except for Zakerifar et al. (2011) who introduce a kriging simulation metamodel which can be used for parameter tuning under multiple objectives.

### Sensitivity analysis

Sensitivity analysis provides information on how the output of a model varies depending on its inputs. Model inputs (factors) can be subdivided into system parameters and control (or design) variables. Comprehensive overviews are given by Iooss and Lemaître (2015), Pianosi et al. (2016). A main goal lies in the determination of robust solutions accounting for parameter uncertainty. It is distinguished between local and global sensitivity analysis; the former/latter considers variation of one/several parameter(s). Borgonovo and Plischke (2016) concludes that sensitivity analysis is a prerequisite to align communication between modelers, analysts, and decision makers leading to a profound system understanding. In the context of simulation models, the design of experiments (DoE) prescribes the experimental outline with respect to the input factor variation according to which model executions are carried out (Kleijnen 2007). DoE is concerned with limiting computational efforts. Montevechi et al. (2010) shows how a full factorial design can be reduced to a fractional design by intermediate screening factor influences. Gutenschwager et al. (2017) distinguishes between static and dynamic factor design; the former prescribes a list of factor combinations to be examined, while the latter allows for dynamic guidance based on sensitivities found previously.

Global sensitivity analysis comprehensively determines single and joint factor influences as it allows for simultaneous variations over the entire parameter space (Iooss and Lemaître 2015). Variance-based sensitivity analysis is rooted in the decomposition of the overall variance breaking it down to components attributable to factor groups (Prieur and Tarantola 2016). Compared to the classical analysis of variance in a factorial design, the Sobol’ method considers input parameters as random variables (Archer et al. 1997). Numerically, Sobol’ indices are computed through Monte Carlo simulation. In the simulation context, Chan et al. (1997) find the Sobol’ method effective to attribute factor contributions to output variability, eliminating insignificant parameters, determining interactions between factor subgroups, and guiding the search for optimal regions of the parameter space. Chen et al. (2005) further recommends to use simulation metamodels to diminish computational efforts. A comparison of the Sobol’ method to other sensitivity analysis techniques is presented in Yang (2011).

### Multi-criteria decision making

Multi-criteria decision making (MCDM) deals with making decisions in the presence of multiple conflicting objectives. Depending on the domains of decision alternatives and their level of measurement, different methods are available. Greco et al. (2016) compiles surveys on different settings resulting from decision and attribute types including, e.g., preference modeling, intangible criteria, utility theory. Extensions to uncertainty are found in Durbach (2014), Mareschal (1986). An overview on multi-criteria optimization for numerical inputs and objectives with interval scale measurement is discussed in Ehrgott (2005) for various solution concepts such as dominance, efficiency, lexicographic ordering, or scalarization. Efficiency concepts for MCDM problems under uncertainty are presented in Abdelaziz et al. (1999). As dealing with uncertainty is intractable for realistic multi-criteria problems, several frameworks connecting simulation and uncertainty are proposed bringing the topic closer to sensitivity analysis. An overview is given in Broekhuizen et al. (2015) suggesting that different types of uncertainty could be examined by simulation-based sensitivity analysis.

Various frameworks combine simulation and MCDM. In Aickelin et al. (2018), simulation guides the multi-criteria analysis dynamically by evaluating the quality of different decision options. A combination of discrete-event simulation, genetic algorithms, Taguchi robustness and utility-function-based MCDM is established in Al-Aomar (2002) to provide robust parameter settings in a multi-objective environment. A simulation-guided approach to conduct a sensitivity analysis of utility function weights of a multi-criteria objective is given by Butler et al. (1997) who employ random-weighting, rank-order weighting, and assessed-weightings in simulation models to infer knowledge about effects of simultaneous weight changes.

Broekhuizen et al. (2015) reviews MCDM under uncertainty in health care by distinguishing different approaches including deterministic and probabilistic sensitivity analysis. Deterministic sensitivity analysis is by far the most popular approach, although for multiple uncertainty sources sophisticated methods are more recommendable. Marsh et al. (2014) surveys different MCDM methods to improve health care interventions. Different possibilities for health technology assessment under multiple criteria are compared in Thokala and Duenas (2012). Jun et al. (1999) point out that due to multiple objectives prevalent in health care, simulation models are particularly viable to decision making.

### Decision support systems

Decision support systems (DSS) are computerized tools and models which assist decision makers to derive “better” decisions. They build upon existing methods like simulation or MCDM and refine them with additional decision-relevant aspects like, e.g., expert opinions, interactive visualization, databases. A historical overview is given by Power (2008). The overview Siskos and Spyridakos (1999) emphasizes the future need for integration of artificial intelligence. Accordingly, recent research is headed in this direction (Delen and Sharda 2008; Li et al. 2010), e.g., through metamodeling by artificial neural networks, kriging, or multivariate adaptive regression splines. From the categorization of DSS into communication-driven, data-driven, document-driven, knowledge-driven, and model-driven DSS (Power and Sharda 2007), we are concerned with the latter one due to the combination of simulation, optimization, and sensitivity analysis. Future research is seen in the integration of behavioral components (e.g., agent-based models) and technological components (e.g., user interfaces). A particular consideration of multiple criteria is outlined in Korhonen et al. (1992) who describe how decision makers can be supported in structuring and solving multi-criteria problems. A framework for integrating uncertainty in trade-offs between different criteria into a DSS is elaborated in Podinovski (1999). Guariso et al. (1996) suggests to build a DSS around an integrated simulation and optimization environment to conduct the optimization of simulation model parameters in a seamless environment. Chatha and Weston (2006) combines discrete-event simulation, systems thinking, and enterprise modeling to obtain an integrated DSS for decision making amidst the complexity of manufacturing organizations. General principles and foundations to be followed in designing DSS based on simulation models are derived in Page (1994). Related to health care, Everett (2002), Kadri et al. (2014b) provide examples dealing with patient flow and patient scheduling, respectively.

### Epidemics simulation

An overwhelming amount of research is available for epidemiology modeling and analysis. As surveyed in Brauer et al. (2008), many extensions are available (e.g., stochasticity, population heterogeneity, or aging) for the most basic model for epidemiology dynamics, the SIR model which subdivides the population into susceptible, infectious, and recovered individuals. Many case studies using extensions of this model are available such as for diseases arising from pathogens in water distribution systems (Fajdek et al. 2019) or anthrax attacks (Chen et al. 2006). In epidemiology, decision making is subsumed under prevention and intervention measures. Ma et al. (2011) emphasize that streamlined methodologies are necessary to grant fast development once an epidemic arises. Consequently, a database-supported high-performance epidemic simulation framework is presented. In a similar fashion, but with focus on the network character, an architecture based on demo- and geographic properties is developed in Huang et al. (2010). An intervention simulation study related to social distancing is given in Kelso et al. (2009). Especially in epidemic simulation studies, data and model assumptions are crucial in terms of decision quality as concluded in Orbann et al. (2017) for data definitions and in Özmen et al. (2016) for model assumptions. Accordingly, simulation-based decision making and sensitivity analysis have to be combined for model validation. However, no unified outline is available and the spectrum of methods is vast. Rao et al. (2009) derive implications through case studies where each one answers a specific research question on the avian influenza outbreak. A sensitivity analysis environment based on Monte Carlo simulations is applied in Ma and Ackerman (1993), Ma et al. (1993) for multiple objectives in viral epidemics (illness attack rates, durations, peaks). Parameter variations are carried out for influenza (Nsoesie et al. 2012) and Ebola epidemics (Legrand et al. 2007) to gain knowledge on root causes and effects. Recent topics of epidemics simulations consider the integration of real world aspects such as mobility patterns (Kopman et al. 2012), integration between epidemics and hospital processes (Nikakhtar and Hsiang 2014), and impacts of epidemics on global supply chains under multiple objectives (Ivanov 2020).

### Research gap and research questions

As shown by the literature review, no comprehensive framework exists for supporting decision makers in setting control variable values in a dynamic system subject to uncertain data and several conflicting objectives. In particular, methods from different related disciplines (such as simulation optimization, sensitivity analysis, or multi-criteria decision making) appear segregated from each other and come with their individual flaws when considered as part of an integrated approach (such as consideration of single objective only, insufficient consideration of data assumptions, or dependence on substitute optimality concepts). Hence, a required initial step for closing this research gap consists of devising a general architecture of a model-based decision support framework assisting in the selection of control variable values in dynamic systems under uncertain system parameter values and multiple objectives. The urgent necessity for such an overarching methodology has become apparent during the outbreak of the pandemic due to the coronavirus in early 2020. As typical for such novel situations, vastly no reliable data is available and information is vague. Hence, for such cases we identify the need for an interactive character of a decision support methodology. Overall, the research questions can be summarized as follows:

- What is the general structure of a framework for model-based decision support in the presence of multiple conflicting objectives and data uncertainty?
- What is an instantiation of this general outline with respect to a specific composition of involved methodological components?
- How can this framework be applied practically?

Section 3 provides answers to the first two questions on a methodological level, whereas the case study on an infectious disease epidemic in Sect. 4 addresses the third question from a practical perspective.

## Simulation-based multi-criteria decision support with sensitivity analysis feedback

Initial phases of a simulation study comprise the statement of the overall mission, specifications of study requirements and required data, as well as the formation of a conceptual and an executable model of the dynamic system (Gutenschwager et al. 2017). The first two steps determine which factors are control variables and which are system parameters. Control variables can be used directly for influencing the system behavior, whereas system parameters influence the system, but cannot be changed in the short term. Uncertain system parameters require an explicit analysis to increase confidence in decisions on control variables.

We introduce a new interactive methodology for the analysis of complex dynamic systems under multiple objectives and parameter uncertainty. The analysis is directed at determining the most favorable values for control variables, accounting for the multi-criteria character and enriching decisions with sensitivity information on system parameters and control variables. While several methods are available and viable for the simulation-based one-dimensional optimization of system parameters or control variables (such as metaheuristics, gradient-descent, simultaneous perturbation; see Gosavi (2015); Amaran et al. (2016)), the case of multiple objectives has experienced considerably less attention yet. As outlined in Sect. 1 and displayed in Fig. 1, the methodology proceeds iteratively and allows the decision maker to interact with the method to cover different topics of interest concerning the system behavior. Each iteration is based on a guiding analysis question, system parameters configuration, and set of control variables configurations to be examined. In each iteration, the following steps can be used in a customized fashion to answer the current analysis question.

1. *Simulation* Variation experiment with control variables: For the current system parameters configuration, collect information on the performance achievable for the current set of control variables configurations with respect to the multiple objectives.
2. *Multi-criteria decision making* Determination of efficient control variables configurations: From all control variables configurations considered in step 1, determine which of them are efficient according to a prescribed criterion of efficiency.
3. *Simulation* Perturbation experiment with system parameters: Perturb the current system parameters configuration from step 1 and record the effect on the multiple objectives resulting from the efficient control variables configurations from step 2.
4. *Sensitivity analysis* Detection of critical control variables and system parameters: Apply a sensitivity measure upon the multi-criteria decision making results from step 2 and the perturbation results from step 3 to identify sensitive control variables and system parameters, respectively.
5. *Update* Re-select the current guiding analysis question, control variables configurations and system parameters configuration for the next iteration based on the sensitivity analysis results from step 4.

**Fig. 2:**
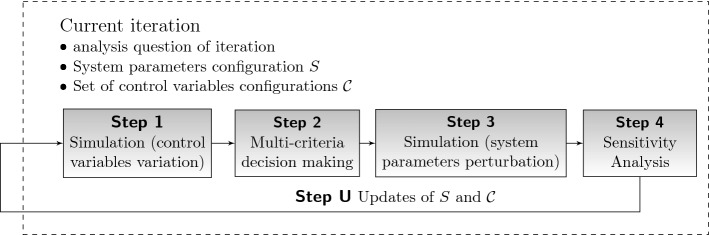
Iterative step in the interactive methodology for simulation-based multi-criteria decision support with sensitivity analysis feedback

Figure 2 illustrates the outline of one iteration. Due to the update step, the overall procedure becomes interactive allowing the decision maker to impute a certain setting of special interest upon each iteration such that steps 1 to 4 can be tailored towards answering the current analysis question. Over the course of the iterations, the decision maker accumulates an information pool representing the acquired knowledge on the system behavior.

After introducing the terminology and notation in Sect. 3.1, details on the individual steps are given in Sects. 3.2 to 3.6. The number of iterations is rather limited as each iteration answers a specific analysis question posed by the decision maker to understand the system more thoroughly. Therefore, whilst steps 1 to 4 are executed in automated fashion, the updating step is implemented in interaction with the decision maker.

### Terminology and notation

We denote a control variables configuration, i.e., a tuple of control variable values, by $C := (c_1, c_2, \ldots , c_{n_c})$ where $n_c \in {\mathbb {N}}$ is the number of control variables and $c_i$ with $i \in \{1, 2, \ldots , n_c\}$ is the value of the *i*th control variable. A set of control variables configurations is referred to by ${\mathcal {C}}$. Likewise, we denote a system parameters configuration, i.e., a tuple of system parameter values, by $S := (s_1, s_2, \ldots , s_{n_s})$ where $n_s \in {\mathbb {N}}$ is the number of system parameters and $s_i$ with $i \in \{1, 2, \ldots , n_s\}$ is the value of the *i*th system parameter. A set of system parameters configurations is referred to by ${\mathcal {S}}$. As a generalization, the value of a factor *f* can either be the value of a control variable or the value of a system parameter. We denote a factors configuration, i.e., a tuple of factor values, by $F := (f_1, f_2, \ldots , f_{n_f})$ where $n_f \in {\mathbb {N}}$ with $n_f \le n_c + n_s$ is the number of considered factors and $f_i$ with $i \in \{1, 2, \ldots , n_f\}$ is the value of the *i*th considered factor. A set of factors configurations is referred to by ${\mathcal {F}}$. The system performance achieved with control variable configuration *C* under system parameters configuration *S* is indicated by the objectives configuration $O(C,S) := (o_1, o_2, \ldots , o_{n_o})$ where $n_o \in {\mathbb {N}}$ is the number of objectives and $o_i$ with $i \in \{1, 2, \ldots , n_o\}$ is the value of the *i*th objective. A set of objectives configurations is referred to by ${\mathcal {O}}$. The objectives configurations obtained by combinations of control variables configurations from set ${\mathcal {C}}$ and system parameters configurations from set ${\mathcal {S}}$ can be recorded in a performance table ${\mathcal {O}}({\mathcal {C}}, {\mathcal {S}})$ consisting of triples (*C*, *S*, *O*(*C*, *S*)) with $C \in {\mathcal {C}}$ and $S \in {\mathcal {S}}$.

### Step 1: Simulation—Variation experiment with control variables

For the current system parameters configuration *S*, the system performance is evaluated over all possible control variables configurations in ${\mathcal {C}}$. Typically, in the first iterations, *S* represents a system parameters configuration which is considered most probable or which represents a configuration of interest; ${\mathcal {C}}$ contains those control variables configurations that comply with the degrees of freedom given to the decision maker for the control variables. To obtain information on the system performance, a simulation model is utilized as a proxy for the dynamic system. The variation experiment then executes the simulation model for each pair (*C*, *S*) with $C \in {\mathcal {C}}$ and records the obtained performance *O*(*C*, *S*) in ${\mathcal {O}}({\mathcal {C}}, \{S\})$. Algorithm 3.1 provides the computational steps to obtain this performance table. With an upper bound $T^{sim}$ on the runtime for a simulation replication, the algorithm runs in $\mathrm {O}(|{\mathcal {C}}| \cdot T^{sim})$ time. We remark that in case of prohibitive computational effort for executing individual simulation replications, simulation metamodels can serve as surrogate models for approximating the simulation outcome in a computationally efficient manner (Barton and Meckesheimer 2006). In contrast to the sensitivity analysis based on perturbations of *S* (cf. step 3), step 1 considers variation of control variables configurations in ${\mathcal {C}}$ in order to collect data upon achievable objectives configurations for given *S*. The performance table ${\mathcal {O}}({\mathcal {C}}, \{S\})$ is the input for the determination of efficient control variables configurations in step 2. 
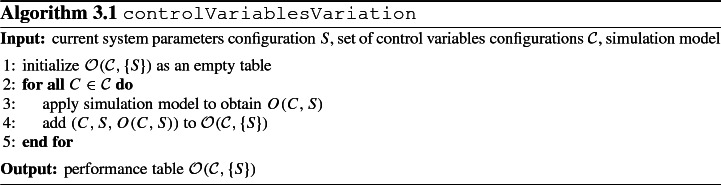


### Step 2: Multi-criteria decision making—Determination of efficient control variables configurations

To cover a wide array of different approaches to multi-criteria decision making, we introduce the general notion of an efficiency criterion. An efficiency criterion is defined as a property of a control variables configuration that can either be fulfilled or not fulfilled. A control variables configuration is called efficient if and only if it fulfills the efficiency criterion. The efficiency criterion can be formulated verbally or mathematically as long as its validity for a control variables configuration can be evaluated computationally.

Step 2 then consists of determining for the current system parameters configuration *S* the set of efficient control variables configurations ${\mathcal {C}}^*(S)$ containing all control variables configurations in the performance table ${\mathcal {O}}({\mathcal {C}}, \{S\})$ fulfilling the efficiency criterion. Algorithm 3.2 summarizes the computational steps for efficiency criterion *E* and performance table ${\mathcal {O}}({\mathcal {C}}, \{S\})$. With an upper bound $T^{eff}$ on the runtime for an efficiency check of a control variables configuration, the algorithm runs in $\mathrm {O}(|{\mathcal {C}}| \cdot T^{eff})$ time. The algorithm output yields those efficient control variables configurations whose sensitivity with respect to variations in control variables shall be considered in step 4 and whose sensitivity with respect to variations in system parameters shall be considered in step 4 after first perturbing the current system parameters configuration *S* in step 3. 
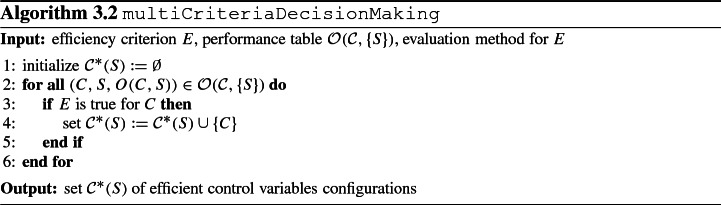


We now instantiate step 2 for the case where the efficiency criterion *E* amounts to Pareto optimality. We emphasize that this just represents one option for determining efficient control variables configurations. Other methods for multi-criteria optimization as presented in Ehrgott (2005); Greco et al. (2016) can be applied similarly. We give the definition of Pareto optimality using the notation introduced previously as follows: A control variables configuration *C* is Pareto optimal with respect to a given performance table ${\mathcal {O}}({\mathcal {C}}, \{S\})$ if and only if there is no control variables configuration $C' \in {\mathcal {C}}$ with $O(C', S)_i \le O(C, S)_i$ for all $i = 1, \ldots , n_o$ and $O(C', S)_i < O(C, S)_i$ for at least one $i \in \{1, \ldots , n_o\}$. Algorithm 3.3 represents the subroutine to check whether *E* is true for control variables configuration *C* under the efficiency criterion of Pareto optimality. Without loss of generality, the algorithm assumes that smaller values are preferred over larger values for each objective. 
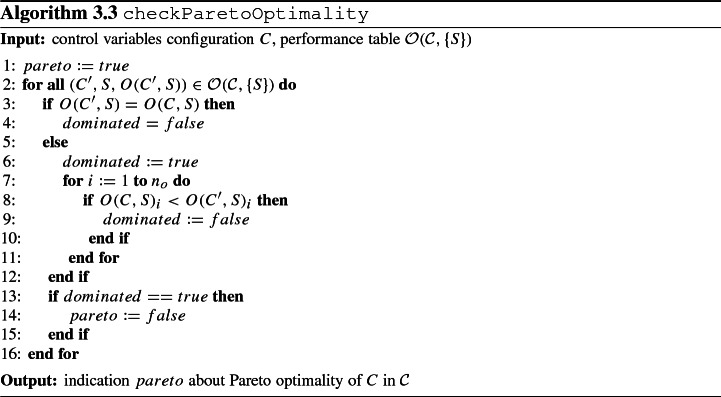


### Step 3: Simulation—Perturbation experiment with system parameters

For each efficient control variables configuration $C \in {\mathcal {C}}^*(S)$, we examine how the objectives configuration *O*(*C*, *S*) changes when up to $n_s$ elements of the current system parameters configuration $S = (s_1, s_2, \ldots , s_{n_s})$ are varied slightly. The set of system parameters configurations which results from perturbing *S* is subsequently denoted by ${\mathcal {S}}(S)$. We record the obtained values of $O(C, S')$ for all $S' \in {\mathcal {S}}(S)$ in performance tables ${\mathcal {O}}(\{C\}, {\mathcal {S}}(S))$. Hence, this step serves as a preparatory data collection step for the sensitivity analysis in step 4. Typically, ${\mathcal {S}}(S)$ shall contain variations in those system parameters of *S* which present themselves to the decision maker with a high degree of uncertainty. As explained in Sect. 3.2, the use of simulation metamodels can be considered in case of substantial computational effort for executing individual simulation replications. As an alternative for the two for all-loops (which can be interpreted as a full factorial design considering the candidate system parameters configurations and the efficient control variables configurations), computational effort in the generation of the array of performance tables ${\mathcal {O}}(\{C\}, {\mathcal {S}}(S))$ with $C \in {\mathcal {C}}^*(S)$ can be diminished through employing an alternative experimental design. Such an experimental design should be capable of providing a suitable representation of the system parameters and/or control variables configuration space (Vu et al. 2016). Algorithm 3.4 outlines the procedure to collect the data subsequently needed for the sensitivity analysis. With an upper bound $T^{sim}$ on the runtime for a simulation replication, the algorithm runs in $\mathrm {O}(|{\mathcal {C}}^*(S)|\cdot |{\mathcal {S}}(S)| \cdot T^{sim})$ time. The array of performance tables ${\mathcal {O}}(\{C\}, {\mathcal {S}}(S))$ with $C \in {\mathcal {C}}^*(S)$ is used in step 4 in order to analyze (in an aggregated form) the sensitivity of the objectives configurations with respect to perturbations in *S* as observed over ${\mathcal {C}}^*(S)$. 
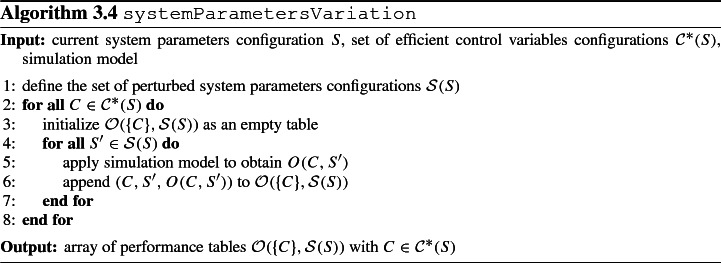


### Step 4: Sensitivity analysis—Detection of critical control variables and system parameters

To obtain sensitivity information on control variables and system parameters, we carry out two types of sensitivity analysis using the results from step 2 (efficient control variables configurations ${\mathcal {C}}^*(S)$) and step 3 (array of performance tables ${\mathcal {O}}(\{C\}, {\mathcal {S}}(S))$ with $C \in {\mathcal {C}}^*(S)$):

**type 1**: Sensitivity of control variables over ${\mathcal {C}}^*(S)$,
**type 2**: Sensitivity of system parameters over ${\mathcal {S}}(S)$ in an aggregated form over ${\mathcal {C}}^*(S)$.

Since—apart from the aggregation for type 2—sensitivity analysis is carried out in the same fashion for types 1 and 2, from now on we commonly refer to a factor which can be either a control variable or a system parameter (cf. Sect. 3.1). Considering the output variability of simulation models, the goal of the sensitivity analysis is to distinguish between influential and non-influential factors as well as to identify interactions between them. 
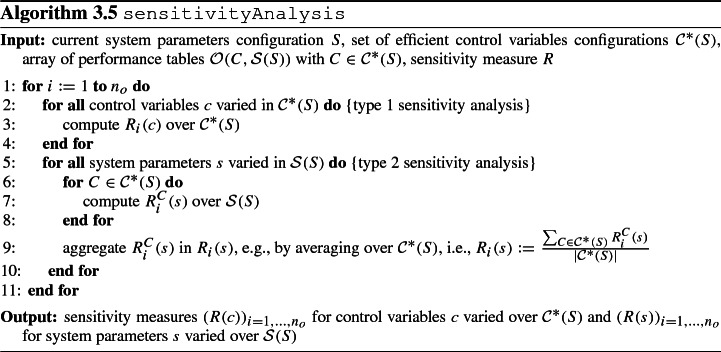


We analyze the sensitivity of a factors configuration *F* over a set of factors configurations ${\mathcal {F}}$. For the sensitivity analysis of type 1, ${\mathcal {F}}$ consists of pairs $F = (C, S)$ with varying $C \in {\mathcal {C}}^*(S)$ and fixed *S*; for the sensitivity analysis of type 2, we first proceed for each $C \in {\mathcal {C}}^*(S)$ individually and afterward aggregate the results. Before aggregation, ${\mathcal {F}}$ consists of pairs $F = (C, S')$ with fixed $C \in {\mathcal {C}}^*(S)$ and varying $S' \in {\mathcal {S}}(S)$.

The decision maker is free to define a sensitivity measure deemed most suitable to convey the sensitivity of *O*(*C*, *S*) upon variation of *C* and/or *S* as prescribed in ${\mathcal {F}}$. A specific sensitivity measure instantiation will be given below in the form of Sobol’ sensitivity indices. Subsequently, we will denote the sensitivity measure of factor *f* by *R*(*f*). The sensitivity analysis is then carried out according to Algorithm 3.5 to capture sensitivities for control variables as found over ${\mathcal {C}}^*(S)$ and for system parameters as found over ${\mathcal {S}}(S)$. Sensitivities are analyzed for each of the $n_o$ objectives on an individual basis. If only a subset of system parameters, control variables, or objectives is of interest, then clearly the computation of sensitivity measures can be restricted accordingly. Resulting sensitivity measures are the basis for deciding upon changes in $S, {\mathcal {C}}$ and the guiding analysis question of the next iteration: When a factor *f* is rather sensitive, then it may be worthwhile to consider a change in *f*; vice versa, when a rather insensitive factor *f* is identified, then the value of *f* can be fixed.

We now instantiate step 4 for the case where the sensitivity measure *R*(*f*) amounts to the Sobol’ sensitivity indices. These indices result from the Sobol’ method as a quantitative global sensitivity analysis method allowing for simultaneous changes in factors (Saltelli et al. 2004; Sobol 1993). We emphasize that this represents one option for determining the model output sensitivity upon changes in factors. Other global sensitivity analysis methods as presented in Iooss and Lemaître (2015); Saltelli et al. (2004) can be applied similarly. For each factor *f*, we consider the first-order Sobol’ index Sob(f) and the total-effect Sobol’ index $\text {Sob}^{\text {t}}(f)$, i.e., we have $R(f) := (R_1(f), R_2(f))$ with $R_1(f) := \text {Sob}(f)$, $R_2(f) := \text {Sob}^{\text {t}}(f)$.

Let $Y := O(F) = g(F)$ be the objectives configuration obtained from running the simulation model with factors configuration *F* where *g* is a (vector-valued) function returning the output of the simulation model under factors configuration *F*. It is easier to understand the following explanations by thinking of *Y* as being scalar-valued and recognizing that the analysis can be adopted for each of the $n_o$ objectives in the multi-criteria setting.

The Sobol’ method is based on the variance decomposition known also from the analysis of variance in classical factorial design. The variance is decomposed into components of increasing dimensionality with respect to the factors involved, hereby reflecting the $2^{n_f} - 1$ non-empty subsets of $F = (f_1, f_2, \ldots , f_{n_f})$. In case of statistically independent factors, *g*(*F*) can be written as a sum of terms $g_i, g_{ij}, \ldots , g_{ij\ldots {n_f}}$ with increasing dimensionality:

$$\begin{aligned} g(F) = g_0 + \sum _{i=1}^{n_f} g_i(f_i) + \sum _{i=1}^{n_f}\sum _{j>i} g_{ij}(f_i, f_j) + \ldots + g_{ijk\ldots {n_f}}(f_i, f_j, f_k, \ldots , f_{n_f}). \end{aligned}$$

Let the expectation and variance of *Y* be denoted by $\text {E}(Y)$ and $\text {Var}(Y)$, respectively. By squaring and integrating, the variance can be computed as

$$\begin{aligned} \text {Var}(Y) = \text {Var}(g(F)) = \int _{K^{n_f}}{g^2(F) dF} - g_0^2 \end{aligned}$$

where $K^{n_f}$ is the ${n_f}$-dimensional space of factors configurations. Hence, in case of mutual independence between factors, *Var*(*Y*) can further be decomposed into a sum of subset variances reflecting interactions between factors of the respective subsets by

$$\begin{aligned} \text {Var}(Y) := D = \sum _{i=1}^{n_f} D_i + \sum _{i=1}^{n_f}\sum _{j>i} D_{ij} + \sum _{i=1}^{n_f}\sum _{j>i}\sum _{k>j} D_{ijk} + \ldots + D_{ijk\ldots {n_f}} \end{aligned}$$

with $$D_i = \text {Var}_{s_i}(\text {E}_{F-\{f_i\}}(Y \,|\, f_i)), D_{ij} = \text {Var}_{f_i, f_j}(\text {E}_{F-\{f_i, f_j\}}(Y \,|\, f_i, f_j)) - D_i - D_j, \ldots $$

The first-order Sobol’ index of factor $f_i$ and the second-order Sobol’ index of factors $f_i, f_j$ are defined as

1 $$\begin{aligned} \begin{aligned} \text {Sob}(f_i)&:= \frac{D_i}{D} = \frac{\text {Var}_{f_i}(\text {E}_{F-\{f_i\}}(Y \,|\, f_i))}{D},\\ \text {Sob}(f_i, f_j)&:= \frac{D_{ij}}{D} = \frac{\text {Var}_{f_i,f_j}(\text {E}_{F-\{f_i, f_j\}}(Y \,|\, f_i, f_j)) }{D} - \text {Sob}(f_i) - \text {Sob}(f_j). \end{aligned} \end{aligned}$$

Sobol’ indices of higher order are defined analogously. In terms of contribution to the total variance, these Sobol’ indices indicate the importance of the *i*th factor, the *i*th and *j*th factor combined, and so on. Note that through normalization by *D* it holds that

$$\begin{aligned} \sum _{i=1}^{n_f} \text {Sob}(f_i) + \sum _{i=1}^{n_f}\sum _{j>i} \text {Sob}(f_i, f_j) + \ldots + \text {Sob}(f_i, f_j, \ldots , f_{n_f}) = 1. \end{aligned}$$

**Fig. 3:**
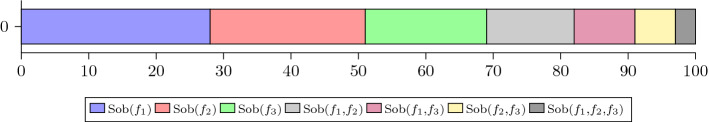
Exemplary illustration of Sobol’ indices for $N=3$ factors

The total-effect Sobol’ index $\text {Sob}^{\text {t}}(f_i)$ gives the overall contribution of factor $f_i$ to the total variance over all possible interactions and it is defined as

2 $$\begin{aligned} \begin{aligned} \text {Sob}^{\text {t}}(f_i)&:= \text {Sob}(f_i) + \sum _{j>i} \text {Sob}(f_i, f_j) + \ldots + \text {Sob}(f_i, f_j, \ldots , f_{n_f}) = 1 - \frac{D_{-i}}{D}\\&= 1 - \frac{\text {Var}_{F-\{f_i\}}(\text {E}_{f_i}(Y \,|\, F-\{f_i\}))}{D} = \frac{\text {E}_{F-\{f_i\}}(\text {Var}_{f_i}(Y \,|\, F-\{f_i\}))}{D} \end{aligned} \end{aligned}$$

with $D_{-i} = \text {Var}_{F-\{f_i\}}(\text {E}_{f_i}(Y \,|\, F-\{f_i\}))$. Observe that the last equation is a direct consequence of the law of total variance. Due to the normalization to 100 % for all Sobol’ indices, the contribution of each factor to the output variability—either due to itself only or due to interaction with other factors—can be illustrated graphically as shown in Fig. 3.

In applications, it is common practice to compute first-order and total-effect Sobol’ indices to evaluate factor sensitivity (Cosenza et al. 2014; Nossent et al. 2011, Quaglietta and Punzo (2013), Song et al. (2012)). In particular, Sobol’ indices can also be utilized in the case of dependent system parameters: According to Saltelli et al. (2004), these measures are still sufficient to rank factors according to importance whilst acknowledging quantitative inaccuracies. Total-effect Sobol’ indices equal to 0 provide a sufficient and necessary condition for the non-influence of a factor. For this reason, statistical software such as *IBM SPSS* use Sobol’ indices in predictor importance algorithms (IBM 2015).

We now illustrate how first-order and total-effect Sobol’ indices can be obtained numerically. For higher-order Sobol’ indices, we refer to Homma and Saltelli (1996). The first part of the discussion deals with the case where Monte Carlo sampling of factors is possible. In this case, a probabilistic model is required to reflect factor combination probabilities. However, in a simulation model for predicting the behavior of a system which is yet unknown even from a probabilistic perspective, sampling according to the distribution of factors is impossible. For this case, we introduce another option which calculates approximations of the Sobol’ indices based on a locally assumed uniform distribution over the factors configurations.

*Probabilistic information available* For the numerical realization through Monte Carlo sampling, observe that factors configurations samples in a sufficient quantity *N* are necessary. A drawback of the numerical method clearly lies in the unavailability of information concerning the suitability of a chosen number of samples and the possibility of excessive computational effort required to execute the simulation model for all samples. In Saltelli et al. (2010), best practices for the computation of $\text {Sob}(f_i)$ and $\text {Sob}^{\text {t}}(f_i)$ are given relying on two matrices of size $N \times {n_f}$ structured as follows:

$$\begin{aligned} A= \left( \begin{array}{cccc} f^{(A,1)}_{1} &{} f^{(A,1)}_{2} &{} \cdots &{} f^{(A,1)}_{{n_f}} \\ \vdots &{} \vdots &{} \vdots &{} \vdots \\ f^{(A,N)}_{1} &{} f^{(A,N)}_{2} &{} \cdots &{} f^{(A,N)}_{{n_f}} \\ \end{array} \right) , \qquad B= \left( \begin{array}{cccc} f^{(B,1)}_{1} &{} f^{(B,1)}_{2} &{} \cdots &{} f^{(B,1)}_{{n_f}} \\ \vdots &{} \vdots &{} \vdots &{} \vdots \\ f^{(B,N)}_{1} &{} f^{(B,N)}_{2} &{} \cdots &{} f^{(B,N)}_{{n_f}} \\ \end{array} \right) . \end{aligned}$$

Each matrix holds *N* factors configurations because each line corresponds to a factors configuration. Matrix *A* is the original sampling matrix holding *N* samples of factors configurations. Matrix *B* can be understood as the basis for resampling, because with *A* and *B*, we can construct ${n_f}$ additional matrices of the form

$$\begin{aligned} AB_i= \left( \begin{array}{cccccc} f^{(A,1)}_{1} &{} f^{(A,1)}_{2} &{} \cdots &{} f^{(B,1)}_{i} &{}\cdots &{} f^{(A,1)}_{{n_f}} \\ \vdots &{} \vdots &{} \vdots &{} \vdots &{} \vdots &{} \vdots \\ f^{(A,M)}_{1} &{} f^{(A,N)}_{2} &{} \cdots &{} f^{(B,N)}_{i} &{}\cdots &{} f^{(A,N)}_{{n_f}} \\ \end{array} \right) \end{aligned}$$

for $i = 1, 2, \ldots , {n_f}$. The simulation model is then carried out for the $N \cdot (2 + {n_f})$ factors configurations as recorded in $A, B, AB_i$ with $i = 1, 2, \ldots , {n_f}$. Denote by $g(A) := \bigl (g(f^{(A,1)}_{1}, \ldots , f^{(A,1)}_{{n_f}})\bigr )_{j=1, \ldots , N}$ the objectives vector found by the simulation model on the *N* factors configurations given through the rows of *A*, and define $g(B), g(AB_i)$ analogously. Then, for instance, the following estimators for $\text {Sob}(f_i)$ and $\text {Sob}^{\text {t}}(f_i)$ can be used according to Saltelli et al. (2010):

$$\begin{aligned} \text {Sob}(f_i) = \frac{\frac{1}{N} \sum _{j=1}^{N} g(A)_j g(AB_i)_j - g_0^2}{\text {Var}(Y)}, \qquad \text {Sob}^{\text {t}}(f_i) = \frac{\frac{1}{2{N}} \sum _{j=1}^{N} (g(A)_j - g(AB_i)_j)^2}{\text {Var}(Y)}. \end{aligned}$$

For $g_0$ and $\text {Var}(Y)$ the estimators $g_0 = \frac{1}{N} \sum _{j=1}^{N} g(F^j)$ and $\text {Var}(Y) = \frac{1}{N} \sum _{j=1}^{N} g^2(F^j) - g_0^2$ can be used from any of the matrices with rows $(F^j)_{j=1, 2, \ldots , N}$.

*Probabilistic information not available* This case occurs when the sensitivity of factors of an unknown system is to be considered and when there is no probabilistic information available for the factors. For control variables, this is natural as the decision maker actively sets control variable values. Yet, the decision maker may be interested in knowing the sensitivity of control variables. To this end, the following outline provides a natural approach to sensitivity analysis not only for system parameters where no probabilistic information is available, but also for control variables in general.

Given empirical data about objectives configurations achieved upon varying a factors configuration in a simulation model, empirical versions of the Sobol’ indices can be computed with Eqs. 1 and 2. Since nothing is known about probabilities for factor realizations, the factor whose sensitivity is to be considered represents a random variable with unknown distribution. Yet, we can specify the support of this random variable in terms of plausible values for the factor. As a general principle, choosing the maximum entropy distribution emulates the state of informational nescience as this approach minimizes the amount of a-priori information in the distribution (Jaynes 1957a, b). The uniform distribution over a set of factors configurations ${\mathcal {F}}$ is the maximum entropy distribution among all distributions with support ${\mathcal {F}}$ (which follows from Langrangian relaxation by the definition of the entropy together with the constraint that the sum over all probabilities equals 1). Hence, computing approximations of Sobol’ indices in this way represents a viable substitute concept. It is based on the absence of probabilistic information and instead requires a specification of the support of factor values for which then locally a uniform distribution is imputed. Algorithm 3.6 summarizes the computation of empirical first-order and total-effect Sobol’ indices for a specific objective and variations in the set of factors configurations ${\mathcal {F}}$. 
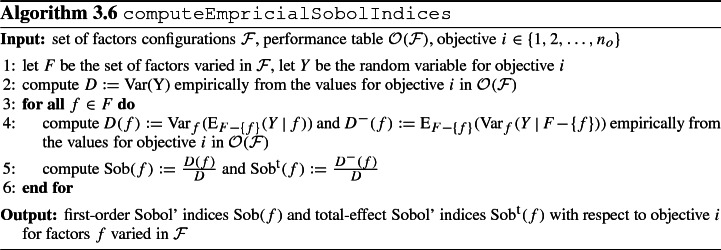


### Step U: Update—Preparation of next iteration

Sensitivity measures $(R(c))_{i=1, \ldots , n_o}$ for control variables *c* varied over ${\mathcal {C}}^*(S)$ and $(R(s))_{i=1, \ldots , n_o}$ for system parameters *s* varied over ${\mathcal {S}}(S)$ yield information on which control variables and/or system parameters are especially sensitive or insensitive. This knowledge is used interactively in the update step as a preparation to answering a new guiding analysis question in the next iteration. This analysis question can be derived from the need of collecting further information upon the system behavior when volatile system behavior has been observed and can be attributed to specific control variables and/or system parameters.

The idea of updating the current system parameters configuration *S* amounts to selecting a new current system parameters configuration of particular interest. It should display changed values for those system parameters $s_i$ ($i \in \{1, 2, \ldots , n_s\}$) which were identified as rather sensitive for at least one of the $n_o$ objectives; system parameters $s_i$ ($i \in \{1, 2, \ldots , n_s\}$) which were identified as rather insensitive for all objectives can be fixed to a reasonable value and be excluded from further consideration. The sensitivity analysis results $(R(s))_{i=1, \ldots , n_o}$ for system parameters *s* varied over ${\mathcal {S}}(S)$ can be added to an information pool representing the decision maker’s knowledge on system parameter sensitivities.

The set of control variables configurations ${\mathcal {C}}$ can be adapted as follows: When efficient control variables configurations result only from a specific value for a control variable $c_i$ ($i \in \{1, 2, \ldots , n_c\}$) or when negligible sensitivity is found for $c_i$, then $c_i$ can be fixed to a reasonable value or restricted to fewer options; a control variable $c_i$ ($i \in \{1, 2, \ldots , n_c\}$) exhibiting a high degree of sensitivity needs further inspection as achieved by increasing the number of potential values for $c_i$ in the updated ${\mathcal {C}}$, e.g., in terms of a finer resolution for $c_i$. The sensitivity analysis results $(R(c))_{i=1, \ldots , n_o}$ for control variable *c* varied over ${\mathcal {C}}^*(S)$ can be added to an information pool representing the decision maker’s knowledge on control variable sensitivities.

We emphasize that there is no standard recipe for updating the guiding analysis question, the current system parameters configuration *S*, and the set of control variables configuration ${\mathcal {C}}$. Therefore, it is recommended to conduct this step interactively with the decision maker upon inspection of sensitivity analysis results and knowledge acquired throughout previous iterations. As a consequence, remaining gaps of knowledge are closed gradually.

## Case study: Simulation-based multi-criteria decision making in infectious disease epidemics

We present the results of a case study on the management of infectious disease epidemics. The goal of the case study is to illustrate how the methodology from Sect. 3 can be used to derive knowledge on ruling mechanisms and principles on the macro-level. Such an outline can be consulted by decision and policy makers to base their decisions on traceable causes-and-effects relations from the simulation-based analysis. We remark that the case study neither claims nor intends to reproduce the real-world dynamics of the 2020 coronavirus pandemic. The dynamic system comprises the population (80 million individuals) of a nation over the course of an infectious disease epidemic (366 days); the goal is threefold: (1) minimize the total number of deaths, (2) minimize the maximum number of critical cases requiring intensive care, (3) minimize economic loss. While the first two objectives directly refer to the number of people reaching the respective states of the dynamic system over the course of the epidemic, the value of the overall economic loss can be computed as the sum of the daily economic losses. Daily economic losses result from the regimes of the economy which are announced by the decision makers. The succession of economic regimes is assumed as follows: (1) full activity, (2) reduced activity, (3) shutdown activity, (4) resumed activity, (5) full activity. For each regime, the corresponding economic loss is estimated as a fixed percentage of the full economic activity which is irreplaceably lost. The system can be controlled by the decision maker by selecting start and end dates of periods of diminished economic activity as well as by selecting the number of intensive care units. The health state of individuals is influenced by epidemiological and medical parameters; economic losses are influenced by economic data. Table 1 summarizes system parameters, control variables, and objectives.

With respect to the modeling paradigm, we take on a system dynamics perspective due to the population size. Hence, information is processed in an aggregated form in terms of average values of population flows over time as opposed to an event- or agent-behavior-based approach which is computationally intractable for millions of inhabitants. In particular, rational behavior of humans cannot be prescribed as opposed to purely industrial settings where agents are expected to obey goal-oriented processing rules (algorithms). Apart from patient zero, each inhabitant starts in a healthy state with respect to the infectious disease. Over the course of the epidemic, a person may become infected and sick. Once this happens, there are three further paths for a person: Direct recovery, indirect recovery (becoming critical first and then recover), or death (becoming critical first and then die). Transitions between states in the form of person flows are driven by the system parameters.

**Fig. 4:**
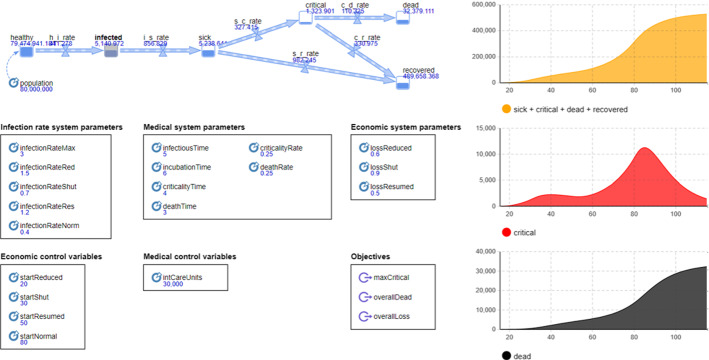
System dynamics simulation model for an infectious disease epidemic in *AnyLogic*

Computational experiments are performed on a computer with Intel Xeon 2.60 GHz processor and 48 GB RAM under Microsoft Windows 10 (64-bit). The system is modeled and executed in the simulation modeling tool *AnyLogic 8.5.2 University Researcher Edition* as illustrated in Fig. 4. Variation and perturbation experiments of the simulation model are implemented using the custom experimentation environment allowing to set control variables and system parameters programmatically as required in each iteration. Interaction with the decision maker is accomplished by prompting the user to enter changes upon control variables and system parameters into the console during the update step after each iteration. Since no optimization algorithms are carried out, runtimes of simulation replications are in the milliseconds allowing for the evaluation of a high number of factors configurations.

**Table 1:** System parameters, control variables, and objectives for the simulation model

System parameters	
*Infection rate data*	
• Initial infection rate under full economic activity (default value: 3 per person, cf. Table 4)	
• Infection rate under reduced economic activity(default value: 1.5 per person, cf. Table 4)	
• Infection rate in shutdown economy (default value: 0.7 per person, cf. Table 4)	
• Infection rate under resumed economic activity (default value: 1.2 per person, cf. Table 4)	
• Final infection rate under full economic activity (default value: 0.4 per person, cf. Table 4)	
*Medical data*	
• Infectious time of an infected person (default value: 5 days, cf. Table 5)	
• Incubation time of the disease (default value: 6 days, cf. Table 5)	
• Time between sickness and criticality (default value: 4 days, cf. Table 5)	
• Time between criticality and death (default value: 3 days, cf. Table 5)	
• Percentage of sick people reaching criticality (default value: 25 %, cf. Table 5)	
• Percentage of critical people reaching death (default value: 25 %, cf. Table 5)	
*Economic data*	
• Economic loss under reduced economic activity (default value: 60 %, cf. Table 6)	
• Economic loss in shutdown economy (default value: 90 %, cf. Table 6)	
• Economic loss under resumed economic activity (default value: 50 %, cf. Table 6)	
Control variables	
*Economic controls*	
• Beginning of reduced economic activity (cf. Tables 2, 3)	
• Beginning of economy shutdown (cf. Tables 2, 3)	
• Beginning of resumed economic activity (cf. Tables 2, 3)	
• Beginning of full economic activity (cf. Tables 2, 3)	
*Medical controls*	
• Number of intensive care units (cf. Tables 2, 3)	
Objectives	
• Minimize total deaths (depending on infection rate data, medical data, economic controls, medical controls)	
• Minimize maximum criticalities (depending on infection rate data, medical data, economic controls, medical controls)	
• Minimize total economic loss (depending on economic data, economic controls)	

### Experimental design

According to the interactive character of the methodology, the case study incrementally improves the decision maker’s understanding of the epidemics dynamics, leading to substantial confidence about decisions on timings of economic regimes and number of intensive care units. The following designs and guiding analysis questions are chosen as a work breakdown.

1. *Focus on control variables* Which control variables are most influential upon system behavior and to what extent?
2. *Focus on critical control variables* How does system behavior change when influential control variables are varied?
3. *Focus on system parameters* Which infection rates are most influential upon system behavior and to what extent?
4. *Focus on system parameters* Which medical data are most influential upon system behavior and to what extent?
5. *Focus on system parameters* Which economic data are most influential upon system behavior and to what extent?
6. *Focus on critical system parameters* How does system behavior change when influential infection rates are changed?
7. *Focus on critical system parameters* How does system behavior change when influential medical data is changed?
8. *Focus on critical system parameters* How does system behavior change when influential economic data is changed?

Observe that this experimental design is pre-specified in advance as it generically covers different elements of the system in the form of control variables and system parameters groups. The interactive component then allows to decide which control variables and system parameters specifically are to be examined in more detail according to the knowledge acquired over previous iterations. We note that another experimental design could start with a single analysis question only, allowing the rest of the experimental design to unfold dynamically.

### Computational results

For the country of Germany with 80 million inhabitants, initial guesses on system parameters are gathered from public discussion and represent the base system parameters configuration as depicted by the values printed in Fig. 4.

#### Iteration 1: Influential control variables

Control variables options for step 1 are pre-specified as shown in Table 2 and executed in a simulation model for any feasible control variables configuration resulting from these values.

**Table 2:** Control variables options and total-effect Sobol’ indices in iteration 1

*Control variables options*	
• Beginning of reduced economic activity	After $\{21, 28, 35, 42\}$ days
• Beginning of economy shutdown	$\{28, 35, 42, 49\}$ days
• Beginning of resumed economic activity	After $\{63, 77, 91, 105\}$ days
• Beginning of full economic activity	After $\{77, 98, 119, 140\}$ days
• Number of intensive care units	$\{25,000, 30,000, 35,000, 40,000\}$ beds
*Control variables sensitivities*	
Total-effect Sobol’ indices for maximum criticalities/total deaths/economic loss	
• Beginning of reduced economic activity	0.73/0.74/0.22
• Beginning of economy shutdown	0.22/0.20/0.07
• Beginning of resumed economic activity	0.01/0.01/0.40
• Beginning of full economic activity	0.00/0.00/0.00
• Number of intensive care units	0.08/0.08/0.30

Step 2 then outputs 29 efficient control variables configurations. All of them exhibit a beginning of full economic activity after 77 days, 25 of them a beginning of resumed economic activity after 63 days. Overall deaths range between 8509 (leading to the worst economic loss) and 43 million individuals (leading to the best economic loss). Clearly, decision makers will not take into account solutions with large death tolls. Therefore, the beginning of the economy shutdown has to be restricted to be no later than 42 days after the beginning of the epidemic, leading to an upper bound of 85,173 deaths. The related maximum number of intensive care units amounts to 22,799.

Step 3 is omitted in the first iteration which focuses on sensitivities of control variables only. In step 4, first-order and total-effect Sobol’ indices over the 29 efficient control variables configurations are calculated for the three objectives. Total-effect Sobol’ indices are summarized in Table 2. In terms of better readability, first-order Sobol’ indices are omitted. Since resumed economic activity begins after 63 days for the most favorable efficient solutions, we find that only the beginning of the reduced economic activity and the beginning of the economy shutdown is worthwhile to be examined in further detail.

From the efficient control configurations, we select the following as the incumbent solution: beginning of reduced economic activity, economy shutdown, resumed economic activity, full economic activity after 21, 35, 63, 77 days, respectively; number of intensive care units of 40,000. The objectives configuration for this control variables configuration amounts to a maximum of 7439 criticalities, 32,080 deaths, and 40.6% economic loss. The same objectives configuration is found for 25,000, 30,000, 35,000 intensive care units. Nonetheless, policy makers are inclined to offer as much intensive care units as possible to prevent emergencies with respect to medical equipment. In particular, with respect to the three objectives considered, there is no influence as long as the number of maximum criticalities is below the lowest option for the number of intensive care units.

#### Iteration 2: Degree of control variables influence

For the most influential control variables found in iteration 1 (beginning of reduced economic activity, beginning of economy shutdown), we increase the number of options for step 1 in both directions as shown in Table 3. Due to minor contribution to objectives variations, the remaining control variables are fixed according to the current favorable efficient solution.

**Table 3:** Control variables options and total-effect Sobol’ indices in iteration 2

*Control variables options*	
• Beginning of reduced economic activity	After $\{17, 19, 21, 23, 25\}$ days
• Beginning of economy shutdown	$\{24, 26, 28, 30, 32\}$ days
• Beginning of resumed economic activity	After 63 days
• Beginning of full economic activity	After 77 days
• Number of intensive care units	$40\,000$ beds
*Control variables sensitivities*	
Total-effect Sobol’ indices for maximum criticalities/total deaths/economic loss	
• Beginning of reduced economic activity	0.79/0.81/0.78
• Beginning of economy shutdown	0.26/0.23/0.17
• Beginning of resumed economic activity	0.00/0.00/0.00
• Beginning of full economic activity	0.00/0.00/0.00
• Number of intensive care units	0.00/0.00/0.00

Step 2 finds 22 efficient control variables configurations with the key insight that in order to keep the number of deaths down it is vital to set both the beginning of reduced economic activity and the beginning of the economy shutdown as early as possible. For instance, if beginnings of reduced economic activity and economy shutdown occur 17 and 24 days, respectively, after the epidemics starts, then the total deaths can be reduced to 2081 with the worst economic loss of 46.3%. Considering the trade-off of an additional economic loss of 5.7% and nearly 32,000 lives saved compared to the solution in iteration 1, decision makers are urged to close the economy as soon as possible. Due to the focus on the control variables configurations, step 3 is omitted. Control variables sensitivities from step 4 confirm the large influence of starting dates of economy closing as seen in Table 3.

We acknowledge that the earliest possible closing options as considered in this iteration are unlikely to be achieved in practice alone by the novelty of the situation and the lack of simulation models which decision makers could have been consulted employed in the midst of a real-world setting coined by a large degree of uncertainty. Additionally, the implementation of measures such as an economy closing takes time and it is arguable whether a full shutdown could have been achieved only one week after the partial closing of the economy.

#### Iteration 3: Influential infection rates

In iterations 3 to 5, system parameter sensitivities are checked group-wise starting with the infection rates. Steps 1 and 2 are first carried out to obtain a set of efficient control variables configurations for the current system parameters configuration as shown in Fig. 4. Table 4 displays the system parameters options which are used in the perturbation experiment in step 3 resulting from perturbing the current system parameters configuration.

**Table 4:** System parameters options and total-effect Sobol’ indices in iteration 3

*System parameters options*	
• Initial infection rate under full economic activity	$\{2.5, 3, 3.5\}$
• Infection rate under reduced economic activity	$\{1.3, 1.5, 1.7\}$
• Infection rate in shutdown economy	$\{0.5, 0.7, 0.9\}$
• Infection rate under resumed economic activity	$\{1.0, 1.2, 1.4\}$
• Final infection rate under full economic activity	$\{0.3, 0.4, 0.5\}$
*System parameters sensitivities*	
Total-effect Sobol’ indices for maximum criticalities/total deaths/economic loss	
• Initial infection rate under full economic activity	0.79/0.79/0.00
• Infection rate under reduced economic activity	0.14/0.18/0.00
• Infection rate in shutdown economy	0.36/0.38/0.00
• Infection rate under resumed economic activity	0.12/0.08/0.00
• Final infection rate under full economic activity	0.00/0.01/0.00

As seen from the total-effect Sobol’ indices in Table 4 (representing averages over all efficient control variables configurations), the initial infection rate under full economic activity and the infection rate in the shutdown economy have the largest impact on objectives variability. This can be explained from the fact that the former infection rate determines the degree of disease spread in the crucial time before economy closings and the latter determines the best case in terms of infection reductions achieved through the shutdown. We will recur to different settings for these two system parameters in iterations 6 to 9 in order to analyze whether previously determined efficient control variables configurations remain efficient upon changes in infection rates.

#### Iteration 4: Influential medical data

For the medically related system parameters, we carry out the same analysis as outlined in iteration 3. Parameter options and sensitivities are summarized in Table 5. From these parameters, the infectious time of an infected person and the incubation time of the disease play a significant role. This becomes clear as the infectious time inversely correlates with the velocity of infection of healthy individuals. On the other hand, the incubation time prescribes how long an infected person remains in the stock of infected individuals representing a threat for healthy people as long as the infectious time is not over. Hence, both times set the outset for the further course of the epidemic situation, although by different mechanisms. Times between sickness and criticality as well as between criticality and death do not matter for the three objectives. This is due to the fact that resulting maximum criticalities and total deaths are not influenced by the amount of time lying between different health states once a person has reached sickness. Trivially, both rates with respect to criticality and death have a direct influence upon criticalities and deaths. However, influences by the infectious time and the incubation time are much stronger in causing changes in total deaths and maximum criticalities as these quantities are in the first place determined by the disease outbreak. We will recur to different settings for these two system parameters in iterations 10 to 13 in order to analyze whether previously determined efficient control variables configurations remain efficient upon changes in infectious times and incubation times.

**Table 5:** System parameters options and total-effect Sobol’ indices in iteration 4

*System parameters options*	
• Infectious time of an infected person	$\{4, 5, 6\}$
• Incubation time of the disease	$\{5, 6, 7\}$
• Time between sickness and criticality	$\{3, 4, 5\}$
• Time between criticality and death	$\{2, 3, 4\}$
• Percentage of sick people reaching criticality	$\{0.15, 0.25, 0.35\}$
• Percentage of critical people reaching death	$\{0.15, 0.25, 0.35\}$
*System parameters sensitivities*	
Total-effect Sobol’ indices for maximum criticalities/total deaths/economic loss	
• Infectious time of an infected person	0.80/0.81/0.00
• Incubation time of the disease	0.54/0.52/0.00
• Time between sickness and criticality	0.00/0.00/0.00
• Time between criticality and death	0.00/0.00/0.00
• Percentage of sick people reaching criticality	0.09/0.08/0.00
• Percentage of critical people reaching death	0.06/0.06/0.00

#### Iteration 5: Influential economic data

For the economically related system parameters, we carry out the same analysis as outlined in iteration 3. Parameters options and sensitivities are summarized in Table 6. From the linear relations between loss levels and control variables (beginning of reduced economic activity, beginning of economy shutdown, beginning of resumed economic activity)—each one resulting in a specific amount of days of an economic regime—each economic loss data demonstrates a significant influence upon the overall economic loss. Clearly, the economic loss parameters do not affect the two medically related objectives. Because of the linear relation, the economic loss objective is well-understood in terms of its dependency on the economic parameters. Thus, a more detailed analysis is not necessary.

**Table 6:** System parameters options and total-effect Sobol’ indices in iteration 5

*System parameters options*	
• Economic loss under reduced economic activity	$\{0.4, 0.6, 0.8\}$
• Economic loss in shutdown economy	$\{0.85, 0.9, 0.95\}$
• Economic loss under resumed economic activity	$\{0.4, 0.5, 0.6\}$
*System parameters sensitivities*	
Total-effect Sobol’ indices for maximum criticalities/total deaths/economic loss	
• Economic loss under reduced economic activity	0.00/0.00/0.31
• Economic loss in shutdown economy	0.00/0.00/0.42
• Economic loss under resumed economic activity	0.00/0.00/0.27

#### Iteration 6–9: Critical infection rates

In the current system parameters configuration of iteration 6 and 7 (8 and 9), we vary the initial infection rate under full economic activity (infection rate in shutdown economy) in the set $\{2.5, 3.5\}$ ($\{0.5, 0.9\}$) as opposed to iteration 1 with a value of 3 (0.7). In each iteration, we inspect the set of efficient control variables configurations to check whether and how the objectives change and whether the currently favorable efficient configuration (beginning of reduced economic activity, economy shutdown, resumed economic activity, full economic activity after 21, 35, 63, 77 days, respectively; number of intensive care units of 40 000) remains efficient. Therefore, steps 1 and 2 are carried out, but steps 3 and 4 are bypassed.

In iterations 6 and 7, efficiency of the incumbent solution is maintained both for infection rates under full economic activity of 2.5 and 3.5. However, as known also from iteration 3, sensitivity is high leading to 3939 deaths and 340,725 deaths, respectively, as opposed to the base case setting with 32,080 deaths. We conclude that the initial infection rate is a crucial quantity with respect to the overall outcome of the epidemic. However, it is evidently difficult to determine precise values for this parameter as it may vary from region to region and also depend on other unknown factors. Clearly, high sensitivity to any uncertain parameter makes decision making under uncertainty inherently difficult as confirmed by the fact that during the 2020 coronavirus pandemic different models came to substantially different conclusions with respect to expected trajectories of the total number of infections and deaths.

In iterations 8 and 9, efficiency of the incumbent solution is maintained both for infection rates in the shutdown economy of 0.5 and 0.9. However, as known also from iteration 3, sensitivity is medium to high leading to 16,944 deaths and 74,025 deaths, respectively, as opposed to the base case setting with 32,080 deaths. Albeit the sensitivity is much smaller than for the infection rate under full economic activity, also the infection rate in the shutdown economy has a major impact on the objectives configuration that will realize in the epidemic. In particular, it is impossible to know the value of this system parameter in advance as its true value realizes during the crisis which is coined by the lack of historic medical data.

#### Iteration 10–13: Critical medical data

Iterations 10 to 13 are carried out following the same outline as discussed for iterations 6 to 9, but with a focus on the two most sensitive medical system parameters (infectious time of a person, incubation time of the disease). In the current system parameters configuration of iteration 10 and 11 (12 and 13), we vary the infectious time of an infected person (incubation time of the disease) in the set $\{4, 6\}$ ($\{5, 7\}$) as opposed to iteration 1 with a value of 5 (6). In all iterations, we find that the currently favorable efficient configuration (beginning of reduced economic activity, economy shutdown, resumed economic activity, full economic activity after 21, 35, 63, 77 days, respectively; number of intensive care units of 40,000) remains efficient. More interesting are the observed changes in the objective values.

We find from iterations 10 and 11 that the infectious time of a person is the most critical system parameter. In case of an infectious time of 4 days under a constant infection rate of 3 infections per person on average, the outbreak velocity explodes leading to 3,231,1134 deaths as opposed to the base case setting with 32,080 deaths. Likewise, in case of an infectious time of 6 days, the disease is under control with 1194 total deaths. The exorbitant sensitivity with respect to the infectious time also explains why in early phases of the 2020 coronavirus pandemic, several models projected millions of deaths. Similar results, albeit not that drastic, are found in iterations 12 and 13 on the incubation time of the disease leading to 6286 deaths in case of an incubation time of 5 days and 139,582 deaths in case of an incubation time of 7 days as opposed to the base case setting with 32,080 deaths. In contrast to the infection rates, uncertainty with respect to incubation times could be reduced through data from hospitalized patients. Similarly, medical research should be directed towards determining the infectious time of a person to reduce uncertainty in future situations.

### Conclusion from computational results

Combining the knowledge from iterations 1 to 13, we resume the following key findings with respect to choosing a most favorable efficient control variables configuration:

- Iteration 1 suggests 29 efficient control variables configurations. A reasonable choice is to set the beginning of reduced economic activity, economy shutdown, resumed economic activity, full economic activity after 21, 35, 63, 77 days, respectively, leading to a maximum of 7439 criticalities, 32,080 deaths, 39.2% economic loss. The available number of intensive care units is not reached. Nonetheless, to hedge against uncertain parameters (as seen in iteration 6 to 13) it may be advisable to select the highest number of intensive care units precautiously.
- Iteration 2 shows that the most critical control variables (beginning of reduced economic activity, economy shutdown) should be selected as early as possible because they pre-determine the further course of the epidemic. Closing the economy partially after 17 days and shutting it down after 24 days could decrease the number of deaths to 2081. However, we acknowledge that quick economy closings may be unrealistic due to the high degree of uncertainty and dynamics in a real world epidemic situation.
- Iterations 3 to 5 analyze the sensitivity of different system parameter groups and find that infection rates, infectious times, and incubation times are most critical. Economic data is uncritical due to the clear effect upon economic overall loss only.
- Iterations 6 to 9 demonstrate that the initial infection rate before any reduction of economical activity is the most sensitive infection rate parameter due to the determining effect on infections during the initial phase of the outbreak.
- Iterations 10 to 13 emphasize the importance for good estimates of infectious times and incubation times. Especially the average infectious time of a person directly determines the velocity of the outbreak, thereby setting the course for the entire epidemic.

Overall, the following recommendations are given for the control variables: the beginning of the reduced economic activity and of the economy shutdown should occur after 21 and 28 days, respectively. If possible, earlier closing actions are beneficial to suppress the crucial initial spread of the disease. Economic activity can be resumed partially after 63 and fully after 77 days because infection events are under control such that further economic loss is avoided due to efficiency considerations. Although uncritical under the system parameters base scenario with the recommended control variables configuration, the number of intensive care units should be selected as high as possible to hedge against parameter uncertainty.

The sensitivity analysis carried out in iterations 1 to 5 and the subsequently refined analysis in iteration 6 to 13 emphasize the strong dependency of the model output on infection rates, infectious times, and incubation times. Hence, the integration of sensitivities into the methodology is an effective way of supporting decision makers in reducing uncertainties as typical for infectious disease epidemics. Difficulties in forecasting the epidemics trajectory arise when critical system parameters are related to properties of a largely unexplored disease. Therefore, the knowledge collected over the iterations helped to gain confidence in the decisions derived over the iterations. Finally, we remark that the observed sensitivities also explain why during the 2020 coronavirus pandemic many models came to different conclusions and some of them excessively overestimated the number of deaths and infections.

## Conclusion and outlook

In this paper, we introduced a comprehensive framework for the simulation-based analysis of dynamic systems under multiple objectives and parameter uncertainty. The methodology is based on a clear distinction between control variables and system parameters as influencing system factors. As a result of the interaction possibilities, decision makers are in a position to examine the system along with factor sensitivities according to a sequence of customized analysis questions over several iterations. Overall, this outline aims at steadily increasing the amount of knowledge that the decision maker possesses concerning the behavior of the system. In a case study on an infectious disease epidemic, the methodology was successfully instantiated in order to determine economically and medically related control variables taking into account sensitivity information on medical and economic objectives. High sensitivity was observed for infection rates, infection time, and incubation time. We believe that the large degree of uncertainty with respect to these parameters plays a major role why during the 2020 coronavirus pandemic different models came to drastically different conclusions. Hence, due to the consideration of sensitivity information, the introduced methodology becomes a reliable tool to hedge against parameter uncertainty arising in complex dynamic systems where causes and effects relationships are still largely unexplored. However, we note that the goal of the case study is not to reproduce the coronavirus pandemic, but rather to give a tool for understanding basic ruling principles of the spread of the disease and to elicit knowledge on the influence of control variables configurations on objectives configurations.

Several directions for future research are suggested: First, integrating model validation would increase the framework reliability, especially when it is unclear whether the simulation model correctly reproduces the real world system behavior. For instance, running one simulation model could be replaced by running a set of simulation models taking into account several possible relations between model entities. A second line of future research regards the use of the framework as an accompanying tool for real world decision making under uncertainty. A systematic approach is needed to adaptively integrate updated corridors for future parameter realizations and to fix past parameter realizations. In this way, the framework and simulation model can be warm-started whenever a decision is required and provide up-to-date decisions. A third line of research represents the use of simulation metamodels as part of the proposed methodology. In case of time-consuming simulation replications (e.g., when operational optimization routines are embedded into the model), metamodeling techniques such as artificial neural networks can be utilized to predict model outputs based on input factors. Finally, apart from the concepts of Pareto optimality and Sobol’ sensitivity indices as presented for steps 2 and 4 in Sect. 3, respectively, further methods for multi-criteria decision making and global sensitivity analysis can be integrated in the framework and assessed in applications to further broaden the scope of the methodology.
